# Removal of Phosphorus from Wastewater by Different Morphological Alumina

**DOI:** 10.3390/molecules25133092

**Published:** 2020-07-07

**Authors:** Jianchuan Sun, Awang Gao, Xuhui Wang, Xiangyu Xu, Jiaqing Song

**Affiliations:** College of Chemistry, Beijing University of Chemical Technology, Beijing 100029, China; 2015400163@mail.buct.edu.cn (J.S.); 2017201058@mail.buct.edu.cn (A.G.); 2016400154@mail.buct.edu.cn (X.W.); xuxy@mail.buct.edu.cn (X.X.)

**Keywords:** adsorption, alumina, phosphorus, high surface area, morphology

## Abstract

In this work, an organic-free method was used to synthesize different morphological boehmite by controlling the crystallization temperature, and alumina adsorbents were obtained by baking the boehmites at 500 °C. The alumina adsorbents were characterized by X-ray diffraction (XRD), High resolution transmission electron microscope (HRTEM), Fourier transform infrared (FT-IR), N_2_ adsorption/desorption analysis, and their phosphorus adsorption properties were comparatively investigated by a series of experiments. The results showed that the self-prepared alumina adsorbents were lamellar and fibrous material, while the industrial adsorbent was a granular material. The lamellar alumina adsorbents had the largest specific surface area and showed better phosphorus adsorption capacity. The maximum adsorption capacity could reach up to 588.2 mg·g^−1^; and only 0.8 g·L^−1^ of lamellar alumina adsorbent is needed to treat 100 mg·L^−1^ phosphorus solution under the Chinese level 1 discharge standard (0.5 mg·L^−1^). Further investigation suggests that the lamellar alumina adsorbent kept high adsorption capacity in various solution environments.

## 1. Introduction

The eutrophication caused by phosphorus pollution is an important issue facing natural water. It has long been concerned by all walks of life, and is especially serious in dense rivers and lakes in some areas. Phosphorus pollution is an imbalance in geochemical balance caused by human social activities over a long time, and exogenous factors lead to phosphorus enrichment in natural freshwater bodies. The eutrophication problem has caused phosphorus-dependent aquatic organisms and microbial abnormal reproduction in coastal waters, lakes, rivers, and the phenomenon of “red tide”. At the same time, the proliferation of phosphorus-dependent organisms consumes dissolved oxygen in the water, causing other organisms to die of oxygen deficiency, seriously damaging the ecological balance in the natural freshwater [1,2].

Various technologies have been developed for removing phosphorus including biological removal process, chemical precipitation, ion exchange and adsorption [3,4,5]. Among them, adsorption is usually regarded as a simple and convenient method. Further research has been done in recent years to search for adsorbent materials suitable for industrial production with high adsorption efficiency [6,7,8,9,10,11,12]. Studies have shown that phosphorus in eutrophic water exists as free phosphate, and a large amount of metal oxide/hydroxide has been used in phosphorus removal because of its specific affinity for phosphate including hydrous ferric oxide [13], magnetite [14], hydrous manganese oxide [15], aluminum hydroxide gel [16], lanthanum hydroxide [17], cerium oxide [18], titanium dioxide [19], hydrous niobium oxide [20], and zirconium oxide/hydroxide [21,22], etc. However, the adsorption capacity of these adsorbents is not high enough (usually <100 mg·g^−1^) and most of them are unsuitable for commercial production due to the complexity of the production process. Thus, it remains a great challenge to prepare high-efficiency and low-cost adsorbents suitable for industrial production for the removal of phosphate in water.

Alumina adsorbent is a cheap material with a broad application foreground in phosphorus removal. However, previous studies have shown that the adsorption capacity of the alumina adsorbent is not high enough because of the low specific surface area [23,24,25,26,27,28]. At present, the industrialized methods for producing alumina include aluminum alcohol hydrolysis, sodium metaaluminate-carbon dioxide method and neutralization method. Among them, the neutralization process is the simplest and the cost is the lowest because it does not use any organic matter and the solutions are easily uniformly mixed [29,30]. However, the specific surface area of alumina prepared by the neutralization method is not high enough for phosphorus removal. In addition, as far as we know, there have been few reports on the application of alumina with a high surface area in phosphorus removal. In this work, the method of neutralization was improved to prepare alumina with a large specific surface area, and alumina adsorbents with different morphologies were obtained by controlling the crystallization temperature. Through a series of adsorption experiments, the performance of alumina adsorbents with different morphologies for phosphorus removal was determined.

## 2. Results and Discussion

### 2.1. Material Characterization

#### 2.1.1. X-ray Diffraction (XRD)

**Figure 1:**
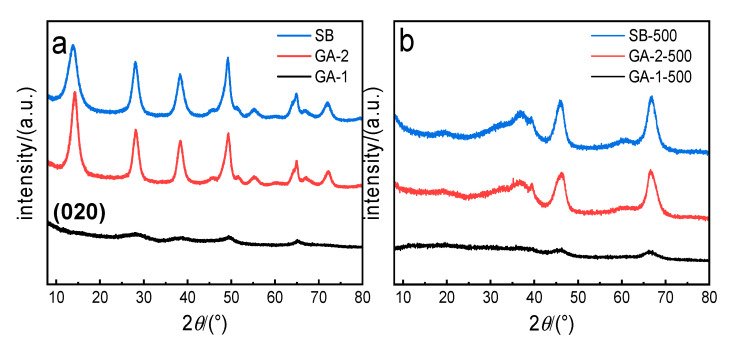
X-ray diffraction (XRD) patterns of (**a**) boehmite and (**b**) alumina adsorbent.

Figure 1a shows the XRD patterns of the as-prepared boehmite samples. All samples showed the typical diffraction of the boehmite phase (JCPDS no.21-1307). The (020) diffraction peak of GA-1 disappeared, implying that the crystalline size along the *b* axis was very small and there was only a single layer of octahedral AlOOH in GA-1 [31]. Figure 1b shows the XRD patterns of the alumina adsorbents obtained from GA-1, GA-2, and SB. According to the standard data (JCPDS card no. 44-1482), all the peaks in Figure 1b were well matched to the characteristic diffraction peaks of γ-Al_2_O_3_ at 19.44°, 37.59°, 45.84°, and 67.00°. The peak intensity of the GA-2-500 adsorbent was consistent with that of SB-500, exhibiting high crystallinity. The peaks of the GA-1-500 alumina adsorbent were broadened to varying degrees, indicating that GA-1-500 has lower crystallinity and smaller crystallites.

#### 2.1.2. High Resolution Transmission Electron Microscope (HRTEM)

**Figure 2:**
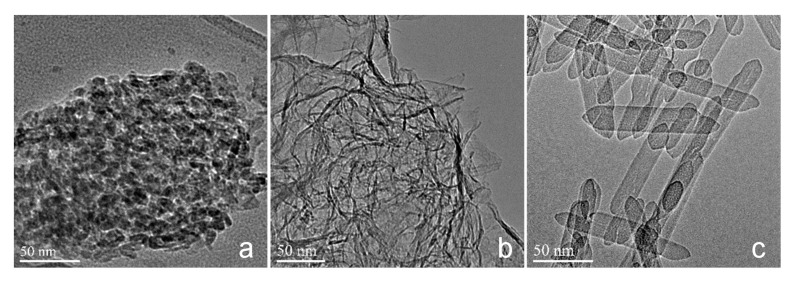
High resolution transmission electron microscope (HRTEM) images of (**a**) SB-500, (**b**) GA-1-500, and (**c**) GA-2-500.

The results of the HRTEM analysis indicated that the three alumina adsorbents had different morphologies. As shown in Figure 2, the industrial adsorbent SB-500 was a granular material (Figure 2a), and the as-prepared alumina adsorbents displayed different morphologies due to different crystallization temperature. The GA-1-500 adsorbent obtained from GA-1 presented a lamellar morphology, and the transition from boehmite to alumina was topological, so the sheet of GA-1-500 was still very thin, which would expose most of the Al to the surface, providing a large number of active adsorption sites. Additionally, GA-2-500 showed a fiber morphology that was more stable at high temperature.

#### 2.1.3. N_2_ Adsorption/Desorption-Analysis

**Figure 3:**
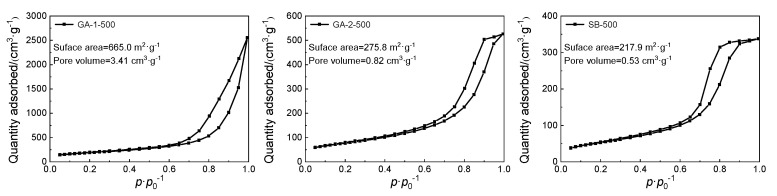
The results of N_2_ adsorption/desorption analysis of alumina adsorbents.

Figure 3 shows the N_2_ adsorption/desorption isotherm curves, specific surface area, and pore volume of the alumina adsorbents. According to the International Union of Pure and Applied Chemistry (IUPAC), all alumina adsorbents showed typical IV isotherms with a hysteresis loop, suggesting these adsorbents were provided with mesopores. The typical IV isotherm has the following characteristics: at lower relative pressure (0 < P/P_0_ < 0.4), the curve is flatter; at higher relative pressure (0.4 < P/P_0_ < 1), the hysteresis loop presents, and represents capillary condensation in mesopores and macropores [32]. A sharp rise in the adsorption volume at high relative pressure was observed in the N_2_ adsorption isotherms of all alumina adsorbents, which suggests the presence of abundant mesopores in these adsorbents. According to the shape of the hysteresis loop, the pore type of the alumina adsorbents can be determined. The hysteresis loops of SB-500, GA-1-500, and GA-2-500 belong to the type of H2, H3, and H2, respectively, suggesting that SB-500 and GA-2-500 have ink-bottle pores and GA-1-500 has cuneiform pores, which is probably formed by lamellar loose accumulation.

As shown in Figure 3, the specific surface area of GA-1-500 was 665.0 m^2^∙g^−1^, which was three times higher than that of SB-500, and was caused by the extremely thin lamellar morphology, while the specific surface area of GA-2-500 was nearly the same as that of SB−500, which is caused by the high crystallization temperature. The pore volume of GA-1-500 was 3.41 cm^3^∙g^−1^ which is 7.4 times greater than that of SB-500, while the pore volume of GA-2-500 was only two times greater than that of SB-500. Due to the difference in morphology, the granular material tended to form close packing, so the thin lamellar material had the largest specific surface area and pore volume, while the granular material had the least specific surface area and pore volume.

#### 2.1.4. Fourier Transform Infrared (FT-IR)

**Figure 4:**
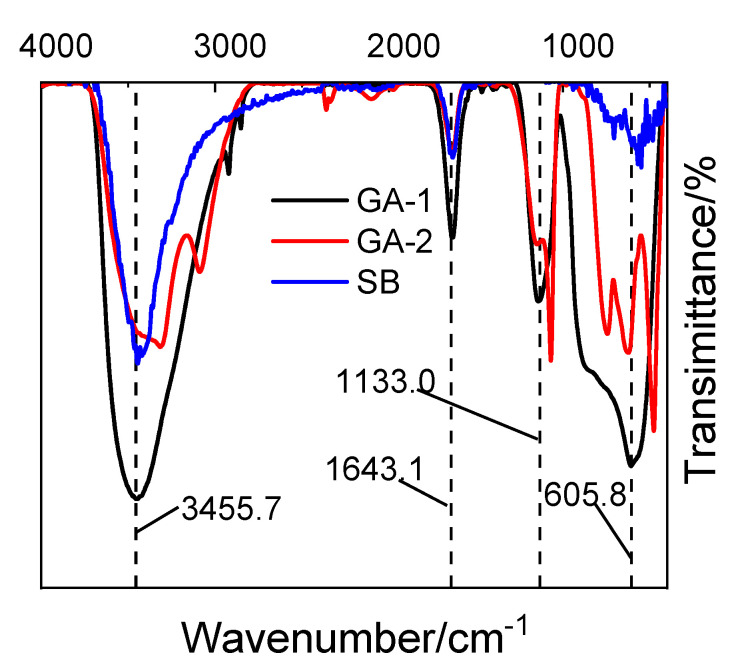
Fourier transform infrared (FT-IR) patterns of different alumina adsorbents.

Figure 4 shows the FTIR patterns of alumina adsorbents. Four absorption bands appeared in the FTIR patterns of the alumina adsorbents. Broad transmittance bands that appeared at 400–1000 cm^−1^ were the characteristic transmittance bands of nano-alumina and the broad transmittance bands appeared at 1000–1600 cm^−1^ were related to the crystal form and the particle size of alumina. All the alumina adsorbents exhibited the hydroxyl group stretching transmittance bands centered at ~3455 cm^−1^ and the peak at 1643 cm^−1^ for the bending vibration of adsorbed H_2_O. It is worth noting that the hydroxyl group stretching bands of GA-1-500 were much stronger than that of GA-2-500 and SB-500, implying that GA-1-500 contains more active hydroxyl group for the phosphorus adsorption. This can be explained by their thin lamellar structures that expose more Al to the surface [33].

#### 2.1.5. Point of Zero Charge

**Figure 5:**
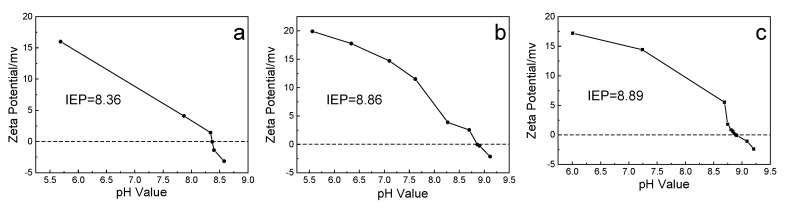
The zeta potential curve of (**a**) GA-1-500, (**b**) GA-2-500, and (**c**) SB-500.

Alumina is amphoteric compounds, and the hydroxyl groups on the surface charge with the change of environmental pH. Under an alkali condition (pH > IEP), the surface hydroxyl groups of alumina dehydrogenate ions to become negatively charged Al–O^−^, while under acidic conditions (pH > IEP), the surface hydroxyl groups of alumina combine with hydrogen ions to become positively charged Al–OH_2_^+^. As shown in Figure 5, GA-1-500 had a lower IEP (8.36) than GA-2-500 (8.86) and SB-500 (8.89), which was caused by the hydrogen ions being weakly dissociated from the hydroxyl group.

### 2.2. Adsorption Behavior

#### 2.2.1. Effect of Adsorbent Dosage

**Figure 6:**
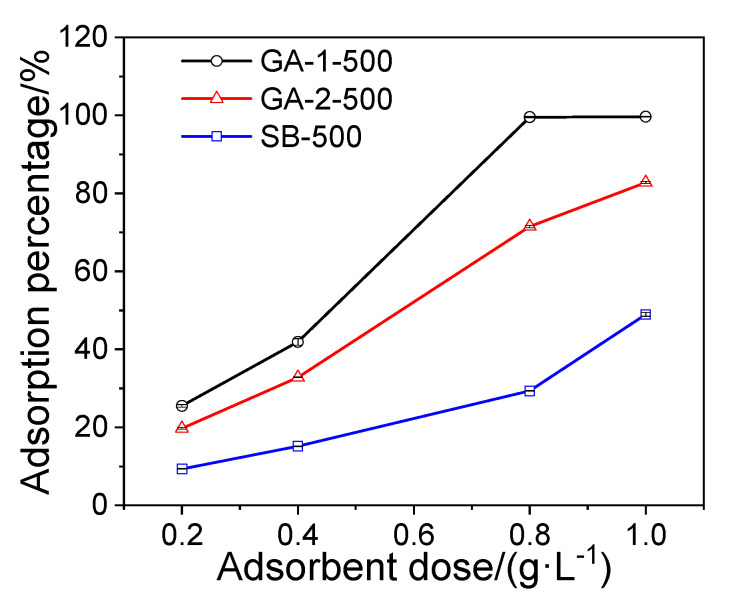
Effect of adsorbent dosage on phosphorus adsorption percentage.

Adsorption behavior is highly dependent upon availability adsorption sites, therefore adsorbent dosage is a significant factor for phosphorus removal. As shown in Figure 6, phosphorus adsorption percentage followed the order of GA-1-500 > GA-2-500 > SB-500 under all adsorbent dosages, suggesting that the morphology of the adsorbent has significant influence on phosphorus adsorption. Thin lamellar GA-1-500 performed much better than the fibrous GA-2-500 and the granular SB-500 adsorbent in phosphorus removal. Only 0.8 g·L^−1^ GA-1-500 was needed to remove 99.6% of 100 mg·L^−1^ phosphorus solution with a residual concentration of 0.4 mg·L^−1^, which is lower than the Chinese level 1 discharge standard (0.5 mg·L^−1^).

#### 2.2.2. Adsorption Kinetics

**Figure 7:**
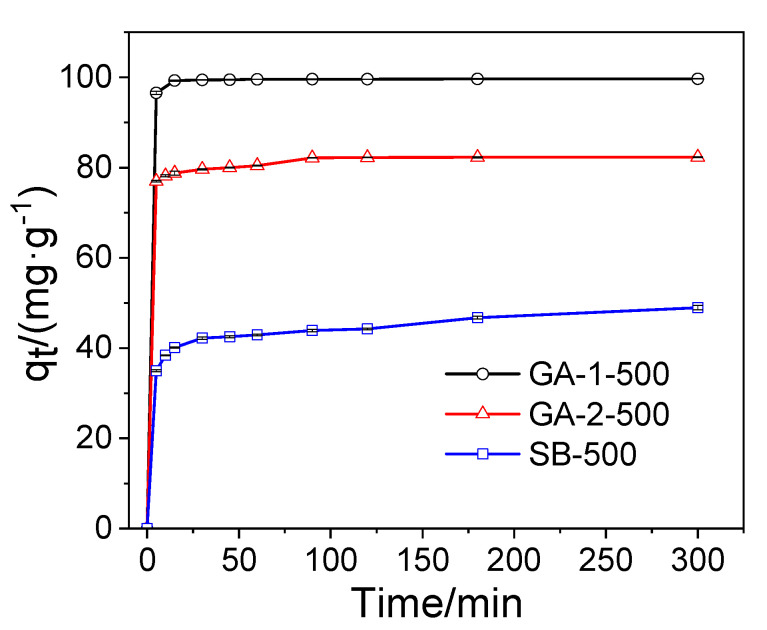
Effect of adsorption time on phosphorus removal.

The effect of adsorption time on phosphorus removal was studied and the results are shown in Figure 7. As shown in Figure 7, the phosphorus adsorption sharply increased during the early adsorption stage, and gradually reached equilibrium. The phosphorus adsorption on GA-1-500 reached the equilibrium state in 60 min with an adsorption percentage of 99.6%. GA-2-500 and SB-500 took a longer time to reach an equilibrium state. It is worth noting that the specific surface areas of GA-2-500 and SB-500 were basically the same, but the adsorption percentage of GA-2-500 (82.3%) was much higher than that of SB-500 (48.9%). This may be because GA-2-500 exposes more crystal surfaces that are conducive to phosphate adsorption than SB-500. In all, the phosphorus adsorption percentage of the adsorbents followed the order of lamellar GA-1-500 (99.6%) > fibrous GA-2-500 (82.3%) > SB-500 (48.9%).

The experimental data were fitted by the pseudo-first-order and pseudo-second-order models, and the fitting results were displayed in Table 1. The pseudo-second-order model provided better agreement between *q*_e,cal_ and *q*_e,exp_ than the pseudo-first-order model, with higher corresponding coefficients (R^2^ > 0.99). This fitting result suggests that phosphorus was removed by alumina mainly via a chemical adsorption process that was due to either the sharing or exchange of electrons as the limiting step, rather than mass transfer. As shown in Table 1, the rate constant (*k*_2_) of the alumina adsorbent followed the order of thin lamellar GA-1-500 (7.03) > fibrous GA-2-500 (1.47) > granular SB-500 (0.34), suggesting that the lamellar adsorbent could remove phosphorus more quickly.

#### 2.2.3. Adsorption Isotherm

**Figure 8:**
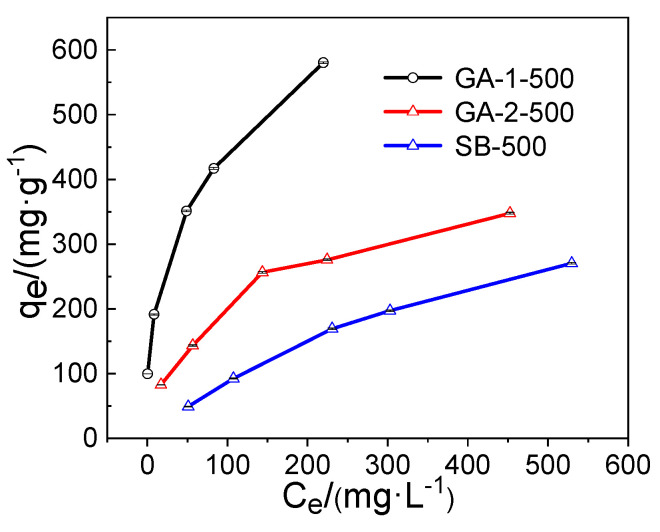
Adsorption isotherm behaviors for different initial concentrations of phosphorus solution.

The results of the adsorption isotherms are presented in Figure 8. It can be seen that the adsorption capacity increased with the growth in the initial concentration of phosphorus. In order to describe the adsorption behavior, the isotherm data were fitted by Langmuir and Freundlich isotherm models and the results are listed in Table 2. The R^2^ values suggest that both isotherm models could well describe the adsorption isotherm of phosphorus removal. The fitting results of the Langmuir isotherm model suggests that the phosphorus removal of alumina adsorbents was a chemical process. It is worth noting that the theoretical saturated adsorption capacity of the adsorbents followed the order of lamellar GA-1-500 (588.2 mg·g^−1^) > fibrous GA-2-500 (360.1 mg·g^−1^) > granular SB-500 (276.5 mg·g^−1^). The theoretical saturated adsorption capacity of GA-1-500 was higher than most of those reported in the literature (Table 3) and the *K*_L_ values of fibrous GA-2-500 and granular SB-500 were smaller than lamellar GA-1-500, which meant that GA-1-500 had the strongest adsorption capacity. The fitting results of the Freundlich isotherm model showed that the 1/n values of self-prepared alumina adsorbents were smaller than 0.5, implying the adsorption process was prone to happen, and the value of lamellar GA-1-500 was obviously smaller than the fiber GA-2-500, which meant that lamellar GA-1-500 could remove phosphorus from wastewater more easily.

#### 2.2.4. Effect of Initial pH and Competitive Ions

**Figure 9:**
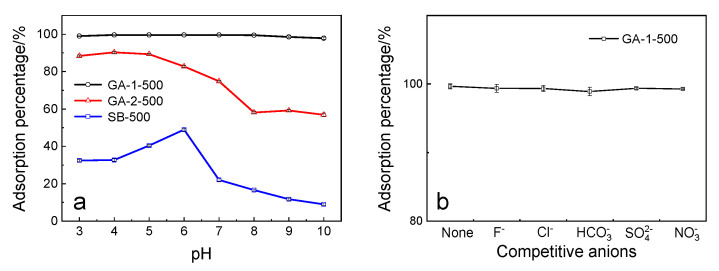
Effect of (**a**) initial pH and (**b**) competitive anions on phosphorus removal.

Figure 9a presents the adsorption behavior of different morphologies alumina adsorbents at various initial pH values ranging from 3.0 to 10.0. The results showed that the adsorption percentage of GA-1-500 maintained a high level (>98%) with the change of initial pH, and other morphology adsorbents showed an obvious fluctuation. For the lamellar GA-1-500, the adsorption percentage was nearly stable, which suggests that a number of active adsorption sites were present at the surface of the material with strong adsorption intensity. At pH > 8, there was a slight decrease in the adsorption percentage, which can be explained by the isoelectric point (IEP). When pH > IEP, the hydroxyl groups were deprotonated, which turns the dominant species of alumina surface to the negatively charged, and this phenomenon was more obvious for GA-2-500 and SB-500.

To investigate the influence of competitive ions on phosphorus removal such as F^−^, Cl^−^, HCO_3_^−^, SO_4_^2−^, and NO_3_^−^, batch experiments were conducted by adding these ions into a phosphorus solution, where the concentration of competitive ions was 100 mg·L^−1^. The results are presented in Figure 9b. It was observed that the adsorption percentage of lamellar alumina was basically not affected by the competitive ions, keeping the value close to 99%. This can be explained by the order of the charge-radius ratio (Z/R) of anion:phosphate > sulfate > chloride > nitrate. The larger the charge-radius ratio, the higher the surface charge density of anion and the stronger the binding ability with the adsorbent.

#### 2.2.5. Adsorption Mechanism

The mechanisms of phosphorus adsorption by an alumina adsorbent have been investigated in previous studies [34,35,36], which could explain that the exchange of hydroxyl group on the surface of aluminum (Al-OH) with phosphorus and formation of phosphorus complex (Al-P) were the main process for phosphorus removal. The process of phosphorus adsorption can be described as hydroxylation that happened on the surface of the alumina in solution, then the hydroxyl group was activated for the positively charged surface; finally, electrostatic adsorption and phosphorus complex formation arose competitively. The difference between the two processes was the stability of the adsorption product; the complex adsorption product was hard to change by other competitive ions, but the electrostatic adsorption product desorbed easily. For GA-1-500, complex adsorption mainly occurred, while complex adsorption of SB-500 and GA-2-500 coexisted with electrostatic adsorption. The adsorption mechanism can be expressed as:

Al-O^−^_(S)_ + 2H_2_O_(l)_ = Al-OH_2_^+^_(S)_ + 2OH^−^_(aq)_ (Surface hydroxylation) Al-OH_2_^+^_(S)_ + H_a_PO_4_^a−3^ = Al-HPO_4(S)_ + a H_2_O_(l)_ + (a−1) OH^−^_(aq)_ (Complex adsorption) AlOH_2_^+^_(S)_ + H_2_PO_4_^−^_(aq)_ = AlOH_2_^+^−H_2_PO_4_^−^_(S)_ (Electrostatic adsorption)

**Figure 10:**
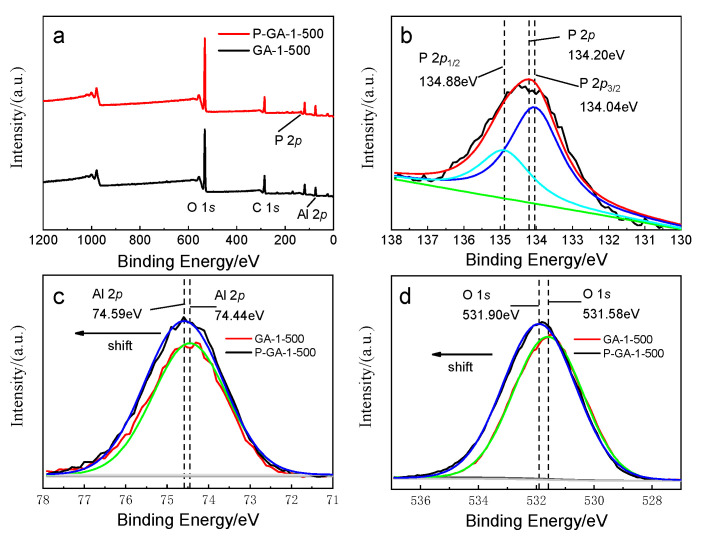
The X-ray photoelectron spectroscopy (XPS) analysis of GA-1-500 before and after phosphorus adsorption. (**a**) XPS full-scan spectra; (**b**) P 2*p* of P-GA-1-500; (**c**) Al 2*p* (**d**) O 1*s*.

In order to clarify the mechanism of phosphorus removal on alumina, XPS analysis was conducted and the results are shown in Figure 10. Distinguished peaks of Al 2*p* and O 1*s* can be observed in the XPS full-scan spectrum of alumina adsorbents. Compared with fresh alumina, the peak attributed to P 2*p* presented in the XPS full-scan spectrum of P-alumina, suggesting phosphorus had been adsorbed onto the surface of alumina. The P 2*p* binding energy peak of P-GA-1–500 was 134.2 eV, higher than the P 2*p* binding energy peak of phosphate, suggesting a formation of strong specific interaction existed between the alumina adsorbent and phosphate [37,38,39,40,41]. The shift of Al 2*p* and O 1*s* binding energy peaks also proves this point.

## 3. Materials and Methods

### 3.1. Synthesis of Adsorbents

Different boehmites as precursors of alumina adsorbents were synthesized via an organic-free neutralization method as reported in the literature [31,42]. Sodium aluminate solution was prepared with aluminum hydroxide and sodium hydroxide at the Na^+^:Al^3+^ molar ratios of 4.5, and the sodium aluminate solution with a concentration of 0.1 mol·L^−1^ was added dropwise into the aluminum sulfate solution under vigorous stirring conditions until the pH reached 9.0, then the suspension was transferred into a Teflon-lined stainless autoclave. After crystallized at a specified temperature for 2 h, the suspension was filtered, washed, and dried at 100 °C overnight to obtain boehmite. The samples crystallized at 90 °C and 170 °C were marked as GA-1 and GA-2, respectively. Alumina adsorbents were obtained by calcining GA-1 and GA-2 at 500 °C for 2 h, which were marked as GA-1-500 and GA-2-500. The commercial boehmite procured from Sasol Germany GmbH (Hamburg, Germany) was marked as SB and the corresponding alumina adsorbent marked as SB-500 was also used to remove phosphorus from wastewater as a contrast sample.

### 3.2. Material Characterization

The X-ray diffraction (XRD) patterns of alumina adsorbents were collected in the 2θ range of 8° to 80° by XRD-6100AS (SHIMADZU, Kyoto, Japan) using Cu-Kα radiation (λ = 0.15406 nm) as a source operating at 40 kV and the scanning speed was 5°∙L^−1^.

The surface area, pore volume, and isotherm adsorption line of the adsorbents were measured by the nitrogen adsorption–desorption method using Quadrasorb (QUANTACHROME, Boynton Beach, FL, USA). The samples were degassed at 300 °C for more than 6 h under vacuum. The liquid nitrogen temperature was 77 K, and high-purity nitrogen was used as the adsorbate.

The morphological properties of alumina adsorbent were analyzed via High resolution transmission electron microscope (HRTEM), using JEM-3010 (JEOL, Tokyo, Japan) with a LaB6 electron gun at 300 kV.

The alumina adsorbents and infrared KBr were dried at 110 °C for 12 h. The adsorbent and KBr were weighed according to a mass ratio of 0.5:100, uniformly ground, and then compressed. FTIR patterns of alumina adsorbents were collected 10 times using ALPHA (BRUKER, Karlsruhe, Germany) and the wavenumber range was 4000–400 cm^−1^, with a precision of 0.01 cm^−1^.

The alumina adsorbents were dispersed into a 0.01 M KNO_3_ solution, and the pH of the system was adjusted with 0.01 M HNO_3_ solution and 0.01 M KOH solution to measure the zeta potential at the corresponding pH, using a ZEN3600 (MAlVERN, Malvern, UK). The isoelectric point (IEP) is the pH of the liquid environment when the surface potential of the adsorbent in a liquid environment is zero.

The alumina adsorbent and the adsorbent after adsorbing phosphate were dried at 110 °C to remove the adsorbed water and then characterized by X-ray photoelectron spectroscopy (XPS) using ESCALAB250 (Thermo Fisher Scientific, Waltham, MA, USA) with monochromatic Al-Kα radiation at voltage of 14 kV and current of 16 mA. Before the test, carbon was contaminated on the surface of the sample, and the binding energy of other elements was calibrated by the binding energy of C 1*S* at 284.6 eV. The obtained data were fitted via XPSPEAK software.

### 3.3. Batch Adsorption Experiments

A suitable amount of KH_2_PO_4_ was dissolved in deionized water to prepare the phosphorus stock solution with a concentration of 1000 mg·L^−1^ (expressed in P), and the phosphorus working solution was prepared via the gradient dilution method. All adsorption experiments were carried out in beakers at 25 °C on a multi-point magnetic stirrer at a shaking speed of 400 r·min^−1^. To identify the effect of the adsorbent dosage on the phosphorus removal, batch experiments were carried out by adding various dosages (in the range of 20 to 100 mg) of adsorbent into 100 mL of a 100 mg·L^−1^ phosphorus solution. To determine the phosphorus adsorption kinetics, 100 mg adsorbent was added into a 100 mL phosphorus solution with a concentration of 100 mg·L^−1^ for various adsorption times ranging from 5 min to 300 min. The phosphorus adsorption isotherm experiments were conducted with the initial phosphorus concentrations of 100, 200, 400, 500, and 800 mg·L^−1^, respectively, and 1 g·L^−1^ of alumina adsorbent was used to adsorb the phosphate solution for 24 h. To study the influence of competitive ions on phosphorus adsorption, 100 mg·L^−1^ Cl^−^, HCO_3_^−^, NO_3_^−^, and SO_4_^2−^ interfering ions were added to a 100 mg·L^−1^ phosphorus solution and these solutions were treated at the adsorbent dosage of 1.0 g·L^−1^. The initial pH of the solutions used above were set as pH 5.0 via 0.1 mol·L^−1^ HCl and 0.1 mol·L^−1^ NaOH. In the pH effect experiments, phosphorus solutions (100 mg·L^−1^, pH = 3–10) were treated with 1 g·L^−1^ of alumina adsorbents for 6 h and the pH value of the phosphorus solution was regulated by 0.1 mol·L^−1^ HCl and 0.1 mol·L^−1^ NaOH.

After the adsorption process, the solutions were filtered using disposable needle filters, then the residual phosphorus concentrations were analyzed by the molybdenum blue method with a visible spectrometer (UV-2600, SHIMADZU, Kyoto, Japan) at 713 nm. The amounts adsorbed at time *t* (*q_t_*, mg·g^−1^) and adsorption percentage (%) of the adsorbents were calculated by the following equations:

(1) $$q_{t}=\frac{V\times (C_{0}-C_{t})}{m}$$

(2) $$\mathrm{Adsorption}\;\mathrm{percentage}=\frac{C_{0}-C_{t}}{C_{0}}\times 100\%$$

where *C*_0_ and *C_t_* mean the initial concentrations and concentrations at time *t* of phosphorus solution (mg·L^−1^), respectively; *V* is the volume of phosphorus solution (L); and *m* is the mass of adsorbent (g). The adsorption kinetic data were fitted by pseudo-first-order and pseudo-second-order models [43,44]. The adsorption isotherm data were fitted by the Langmuir [45] and Freundlich [46] isotherm models.

## 4. Conclusions

In this paper, different morphologies of alumina adsorbents with the morphology of lamellar and fibrous were prepared and a commercial alumina adsorbent with granular morphology was used as a comparison sample. These three morphologies alumina were used to remove phosphorus from wastewater. The lamellar alumina adsorbent exhibited the large surface area of 665.0 m^2^·g^−1^ and showed a high phosphorus adsorption capacity of 588.2 mg·g^−1^, larger than that of fibrous (360.1 mg·g^−1^) and granular (276.5 mg·g^−1^) adsorbents. Further research found that the lamellar alumina showed better absorption efficiency than the fibrous material and granular material in terms of adsorbent dosage and working pH range. The results of the kinetic study and adsorption isotherm study indicated that phosphorus was removed by alumina mainly via a strong chemical adsorption process, which was due to either the sharing or exchange of electrons as the limiting step. Overall, among the three kinds of different morphology of alumina adsorbents, the lamellar alumina showed the best phosphorus adsorption performance.

## Figures and Tables

**Table 1:** Kinetic model parameters of phosphorus adsorption on alumina adsorbents.

	Parameter	SB-500	GA-1-500	GA-2-500
Pseudo-first-order $\frac{dq_{t}}{dt}=k_{1}\left(q_{e}-q_{t}\right)$	*q*_e,exp_ (mg·g^−1^)	48.9	99.6	82.3
	*q*_e,cal_ (mg·g^−1^)	14.6	2.6	11.3
	*k*_1_ (10^−2^·min^−1^)	1.13	3.04	3.998
	R^2^	0.660	0.588	0.852
Pseudo-second-order $\frac{dq_{t}}{dt}=k_{2}{\left(q_{e}-q_{t}\right)}^{2}$	*q*_e,cal_ (mg·g^−1^)	49.0	99.7	82.6
	*k*_2_ (10^−2^·g·mg^−1^·min^−1^)	0.34	7.03	1.47
	R^2^	0.998	0.999	0.999

where *t* is the adsorption time (min); *q*_e_ and *q*_t_ are the amounts adsorbed at the equilibrium state and at given time *t*, respectively (mg·g^−1^); *k*_1_ (min^−1^) and *k*_2_ (g·mg^−1^·min^−1^) represents the rate constants of the pseudo-first-order and pseudo-second-order kinetic adsorption, respectively; *q*_e,exp_ and *q*_e,cal_ is the experimental and calculated values of *q*_e_.

**Table 2:** Adsorption isotherm constants for phosphorus removal by alumina adsorbents.

Kinetic Model	Parameter	SB-500	GA-1-500	GA-2-500
Langmuir isotherm model $q_{e}=\frac{q_{m}K_{L}C_{e}}{1+K_{F}C_{e}}$	*q*_m,cal_ (mg·g^−1^)	276.5	588.2	360.1
	*K*_L_ (L·mg^−1^)	0.0021	0.0468	0.0112
	R^2^	0.998	0.969	0.990
Freundlich isotherm model $q_{e}=K_{F}c_{e}^{1/n}$	1/n	0.75	0.27	0.45
	*K*_F_ ((mg·g^−1^)(L·mg^−1^)^1/n^)	2.71	124.88	23.51
	R^2^	0.992	0.981	0.980

where *C*_e_ (mg·L^−1^) and *q*_e_ (mg·g^−1^) are the concentration of adsorbate in aqueous solution in equilibrium state and the amount of adsorbed adsorbate at equilibrium, respectively; *q*_m_ (mg·g^−1^) denotes the theoretical saturated monolayer adsorption capacity for the adsorbent calculated by the Langmuir isotherm model; *K*_L_ (L·mg^−1^) represents the Langmuir constant; and *K*_F_ and 1/n are the characteristic constants of the Freundlich isotherm, which are the measurements of adsorption capacity and adsorption intensity, respectively.

**Table 3:** Comparison of the phosphorus adsorption capacities of different adsorbents.

Adsorbent	Initial Concentration (mg·L^−1^)	Surface Area (m^2^∙g^−1^)	Experimental Condition	*q*_m_ (mg·g^−1^)	Reference
Alumina	5–200	––	pH < 5.0, 25 °C	20.88	[7]
Ca-Alumina	5–50	348.8	pH = 6.0, 25 °C	8.74	[8]
Aluminum hydroxide gel	41–368	107.2	pH = 3.0–9.0, 25 °C	11.5	[9]
Lanthanum Carbonate	10–200	19.7	pH = 5.0, room temperature	312.5	[10]
Mg-loaded Biochar	20–350	100.0	pH = 8.9, 25 °C	31.2	[11]
Lanthanum/Aluminum Hydroxide Composite	80	99.3	pH = 4.0, 25 °C	76.3	[12]
Alumina GA-1-500	100–800	665.0	pH = 5.0, 25 °C	588.2	This study
